# A Bead-Based Multiplex Assay for the Detection of DNA Viruses Infecting Laboratory Rodents

**DOI:** 10.1371/journal.pone.0097525

**Published:** 2014-05-16

**Authors:** Daniela Höfler, Werner Nicklas, Petra Mauter, Michael Pawlita, Markus Schmitt

**Affiliations:** 1 Research Program Infection and Cancer, German Cancer Research Center, Heidelberg, Baden-Württemberg, Germany; 2 Microbiological Diagnostics, German Cancer Research Center, Heidelberg, Baden-Württemberg, Germany; Charité-University Medicine Berlin, Germany

## Abstract

The Federation of European Laboratory Animal Science Association (FELASA) recommends screening of laboratory rodents and biological materials for a broad variety of bacterial agents, viruses, and parasites. Methods commonly used to date for pathogen detection are neither cost-effective nor time- and animal-efficient or uniform. However, an infection even if silent alters experimental results through changing the animals’ physiology and increases inter-individual variability. As a consequence higher numbers of animals and experiments are needed for valid and significant results. We developed a novel high-throughput multiplex assay, called rodent DNA virus finder (rDVF) for the simultaneous identification of 24 DNA viruses infecting mice and rats. We detected all 24 DNA viruses with high specificity and reproducibility. Detection limits for the different DNA viruses varied between 10 and 1000 copies per PCR. The validation of rDVF was done with DNA isolated from homogenised organs amplified by pathogen specific primers in one multiplex PCR. The biotinylated amplicons were detected via hybridisation to specific oligonucleotide probes coupled to spectrally distinct sets of fluorescent Luminex beads. In conclusion, rDVF may have the potential to replace conventional testing and may simplify and improve routine detection of DNA viruses infecting rodents.

## Introduction

Even silent infections of laboratory rodents with a broad variety of DNA viruses can affect research results. Microorganisms might change the animals’ physiology such as behaviour, growth rate, relative organ weight as well as antibody response or tumour growth [1]–[3]. The impact of infections on experimental data may lead to misinterpretation of results and may be responsible for lack of reproducibility. Consequently, an increased number of animals must be used per experiment to obtain valid and significant data [4]. Therefore, standardised laboratory animal health monitoring is a prerequisite and recommended by international organisations such as FELASA [5]. Although the first FELASA recommendations have been published already 19 years ago [6], there is no uniform program available nor performed in each institute. Thus, a comparison of new assays can only be done with established tests used in an institute performing routine health monitoring.

The prevalence of DNA viruses in laboratory rodents varies between viral species. As shown in several serological studies, mouse parvovirus (MPV) or MPV related strains are most frequently detected in laboratory mice, and rat minute virus (RMV), Kilham rat virus (KRV) and Toolan’s H1 parvovirus (H-1 PV) in laboratory rats [7]–[10]. In an American publication the mouse adenovirus (MAdV) has additionally been reported [11]. Despite improvements of rodent housing and testing, the prevalence of different agents, such as parvoviruses, remained the same or even increased [8]. Furthermore, globalisation and the increasing exchange of genetically modified rodents between research institutions facilitate the spreading of infections. Tests should be able to detect agents in all sources possibly responsible for their introduction into an animal facility. Besides the animals themselves, also biological materials including embryos, sera, monoclonal antibodies, cell lines, cell culture products, tissues and transplantable tumours might be the source of DNA viruses and, thus, should be checked on a regular basis [5], [12].

So far, mainly serological tests are used for the detection of virus infections during routine health monitoring of laboratory animals [13]. Serum samples can be shipped easily, and testing can be performed fast and cheap. However, serological tests are limited to the analysis of serum from immunocompetent sentinel animals. They do not give information if an agent is still present in an animal and if it is still infective. For the detection of contamination in biological materials occasionally still the mouse/rat antibody production (M/RAP) test is applied [14], [15] although PCR tests have been developed [16]–[21]. This test requires the use of animals, is time consuming and therefore expensive. Material is injected into serologically negative mice/rats, followed by an exposure time to allow the rodents to seroconvert. Three to four weeks after the initial exposure, serum samples are collected and analysed by serology [15]. However, MAP test protocols vary among different laboratories [22]. The number of animals tested differs and time allowed for an immune response varies between 21 and 30 days.

Detection of rodent DNA viruses by nucleic acid amplification tests, such as polymerase chain reaction (PCR) is considered to be more sensitive than conventional methods [13], [22]–[25]. A large number of different in-house PCR methods have been described for the detection of DNA viruses included into the FELASA recommendations [16]–[21]. The main disadvantage certainly remains that these assays usually detect only a single type or a small group of rodent viruses. Thus, they fail to assess numerous viruses simultaneously.

However, commercial high-throughput tests directly detecting DNA viruses, e.g. PRIA (Charles River Laboratories, US) are available [26]. Here, we describe a multiplex PCR followed by bead-based Luminex hybridisation as an attractive approach to solve these limitations of in-house PCR. Over the last years, we have already developed several multiplex PCR followed by bead-based Luminex detection using specific probes to detect multiple agents simultaneously in a high-throughput fashion, e.g. human papillomaviruses [27], [28], bovine papillomaviruses [29], human polyomaviruses [30], adeno-associated viruses [31] and cell culture contaminations [32].

Here, we describe a novel high-throughput multiplex assay, called rodent DNA virus finder (rDVF) for the simultaneous detection of 24 rodent DNA viruses infecting laboratory rodents.

## Materials and Methods

### Ethic Statement

The study was approved by the local governmental authorities (Regierungspräsidium Karlsruhe) under the notification number A-25/10.

### Animal Material

Since laboratory animals are rarely infected with DNA viruses, 20 mice and 16 rats with a potentially higher infection rate were bought from 12 pet shops in Germany in order to validate the rDVF assay. Euthanasia was carried out by collecting blood by cardiac puncture in a deep anaesthesia with CO_2_, and death was ensured by placing animals in a prefilled CO_2_ chamber. Of the mice, organs were sampled including salivary gland, ileum, mesenteric lymph nodes, caecum, lung, spleen, trachea, liver, kidney and stomach. However, not every organ of each mouse was available for the validation experiment. In addition, ante-mortem material including faecal samples and pharyngeal swabs were available from 2 and 15 mice, respectively. Altogether, 147 murine samples were accessible.

The following organs were removed from six rats: salivary gland, ileum, mesenteric lymph nodes, caecum, lung, spleen, trachea, liver, kidney, urinary bladder, thymus, brain, and Harderian gland. Ante-mortem material including faecal samples and pharyngeal swabs were taken from all 16, genital and nose swabs from 14 and dermal swabs from six rats. Altogether 170 rat samples were collected.

### DNA Preparation

Tissue biopsies of 10 mg and the collected faecal samples and swabs were homogenised in 650 µL ATL buffer using the TissueLyser II from Qiagen, Hilden (2×2 min at 20 Hz). As negative extraction control, water samples were included. After centrifugation for 3 min at 13000 rpm, DNA was automatically isolated from the supernatant with the QIAsymphony SP instrument using the DSP virus/pathogen mini kit and the complex 400 protocol (Qiagen, Hilden, Germany). The 50 µL eluate was stored at −20°C.

### Multiplex Rodent DNA Virus Finder Assay (rDVF)

Multiplex PCR was performed in a final reaction volume of 25 µL comprising 1x Multiplex PCR Kit buffer (Qiagen, Hilden, Germany), containing 3 mM MgCl_2_, dNTP mix, 0.5×Q-solution and HotStartTaq DNA polymerase, 0.2 to 0.4 µM of each primer, and 1 µL of purified DNA. A 15 min enzyme activation step at 95°C was followed by 45 cycles of amplification in a Mastercycler (Eppendorf, Hamburg, Germany). Each cycle included a denaturation step at 94°C for 30 s, an annealing step at 61°C for 90 s, and an extension step at 72°C for 60 s. The final extension step was prolonged for further 10 min and reactions were kept at 4°C. The detection of amplicons was performed via hybridisation reaction, adding 10 µL of rDVF PCR products to the bead mixture. Next, heat denaturation, hybridisation under stringent conditions, and incubation with streptavidin-R-phycoerythrin (Molecular Probes, Leiden), followed by Luminex read-out, resulted in median fluorescence intensity (MFI) values per target for each specimen.

For each probe, MFI values in reactions with no PCR product added to the hybridisation mixture were considered as background values. Net MFI values were computed by subtraction of 1.2 times the median background value plus 10 MFI. All samples were applied in duplicates. Samples were defined as positive if the net MFI values in both duplicates were above cutoff, and one monitored animal was scored positive if at least one site (organ, ante-mortem sample) was positive for the respective virus.

### Cloning

To determine the assay specificity and sensitivity, plasmids containing the viral target sequences were generated: Purified amplicons were ligated into pSC-B amp/kan vector (4.3 kb) using the StrataClone Blunt PCR Cloning Kit, transformed to competent bacterial cells with heat shock and grown on Ampicillin and X-β-galactosidase coated agar plates. Plasmid-DNA from one clone picked with toothpick and shaked in LB-medium containing Ampicillin overnight was isolated with the Mini-preparation QIAprep Spin Miniprep Kit according to the manufacturer’s instructions. Plasmid DNA was eluted with 50 µL water and stored at 4°C. The viral copy number per unit mass was calculated by assuming that 1 bp weighs about 660 Da. Concentration of the plasmid-DNA was measured with the NanoDrop 1000. Knowledge of the concentration of the purified DNA preparations allowed computing the number of plasmid DNA per µL.

### Sequence Analysis

Purified DNA was amplified using virus-specific primers. For this, a singleplex PCR protocol was employed using the same PCR conditions as for the multiplex PCR. Complementary T3 primer sequences (AATTAACCCTCACTAAAGGGA) were added to the forward or reverse primers. The PCR products were submitted to GATC Biotech (Konstanz, Germany) for sequencing using T3 primer. The nucleotide sequences were aligned using ClustalW2 for identification [33].

### Singleplex Standard PCR

For the confirmation of serological results, the Microbiological Diagnostic Laboratory at DKFZ applied conventional PCR for minute virus of mice immunosuppressive variant (MVMi) and MAdV-1 using the Taq PCR core Kit from Qiagen as recently described with some modifications [34], [35]. Amplicon detection was done by gel-electrophoresis.

Briefly, the parvovirus singleplex PCR (MVMi) comprised a mastermix volume of 46 µL with two family-specific primers in a final concentration of 0.2 µM, 5 µL of 5×PCR buffer, 1 µL of dNTP (10 mM) and 2.5 µL of a fast start high fidelity enzyme (1 U/µL) (Roche Applied Science, Germany) per reaction. Four µL of cell culture supernatant containing MVMi was added. A 30 s denaturation step at 95°C was followed by 35 cycles of amplification in a Mastercycler (Eppendorf, Hamburg, Germany). Each cycle included a denaturation step for 30 s at 95°C, an annealing step for 30 s at 57°C and an extension step for 1 min at 72°C. The final extension was prolonged for further 7 min and reactions were stored at 4°C. The amplified PCR fragment was approximately 310 bp long.

The MAdV-1 singleplex PCR setting comprised a mastermix in a volume of 20 µL with a final primer concentration of 0.2 µM, 2 µL of 10×PCR buffer, 0.5 µL of dNTP (10 mM) and 1.5 µL of Taq polymerase (1 U/µL) (Qiagen, Germany) per reaction. A 5 min activation step at 95°C was followed by 31 cycles of amplification in a Mastercycler (Eppendorf, Hamburg, Germany). Each cycle included a denaturation step for 1 min at 94°C, an annealing step for 1 min at 52°C and an extension step for 1 min at 72°C. The final extension was prolonged for further 7 min and reactions were stored at 4°C. The PCR fragment was 281 bp long.

### Enzyme-linked Immunosorbent Assay (ELISA)

Sera from 20 mice and 14 rats from pet shops were analysed with coated ELISA plates purchased from Charles River (Wilmington, Mass, USA), and tests were performed according to the manufacturer’s instructions. Briefly, 50 µL of the test sera (diluted 1∶60 in 5% skim milk powder solved in PBS pH 7.4) were added to 2 wells of the microtiter plates. One well was used for the antigen test, the other as tissue control. Sera were incubated at 37°C for 40 min. After three washing steps diluted conjugate (horseradish peroxidase-conjugated anti mouse or anti rat IgG) were added to each well. The plate was washed again after 40 min incubation at 37°C, and 100 µL of 0.4 mM ABTS chromogenic substrate was added and incubated for 40 min at room temperature. Colour intensity was measured at 405 nm with an ELISA reader. The net absorbance values were calculated and converted to scores as suggested by the manufacturer. A net score of three or above was considered positive, a score between two and three was considered equivocal.

### Statistics

The coefficient of variation (CV) was computed to describe assay reproducibility with the following equation: CV (%) = 100×standard deviation/mean value. The agreement of ELISA and rDVF was monitored by kappa statistic (κ), where a value of one represents complete agreement, zero represents no agreement.

## Results

### Design of rDVF Assay

One multiplex PCR amplified 24 rodent DNA viruses by employing species- or family-specific primers (Table 1). For internal DNA quality control, the mouse glycerine-aldehyddehydrogenase (gapdh), rat beta-globin (bg) and rodent DNA polymerase (polA) were co-detected. The detection of amplicons was performed via hybridisation to specific oligonucleotide probes coupled to spectrally distinct sets of fluorescent Luminex beads in a single hybridisation reaction. For the detection of related but so far unknown parvovirus species, universal probes were integrated.

**Table 1:** Detection limits (DL) of rDVF for rodent DNA viruses.

Family	Genus	Species	Abbreviation	Host	DL [# of copies/PCRin 100 ng/µLmurine DNA]^a^
Herpesviridae	Rhadinovirus	*Rattus rattus* rhadinovirus 1–3	RratRHV	rat	1000
		*Rattus norvegicus* rhadinovirus 1–2	RnorRHV	rat	10
		*Mus musculus* rhadinovirus 1	MmusRHV	mouse	10
		Murid herpesvirus 4	MuHV-4	mouse, rat	10
	Cytomegalovirus	*Rattus rattus* cytomegalovirus 1	RratCMV	rat	10
		Rat cytomegalovirus Maastricht strain	MuHV-2	rat	100
		Rat cytomegalovirus England strain	MuHV-8	rat	10
		Murine cytomegalovirus	MCMV	mouse	10
Poxviridae	Orthopoxvirus	Ectromelia	ECTV	mouse	100
Adenoviridae	Mastadenovirus	Mouse adenovirus 1	MAdV-1	mouse, rat	10
		Mouse adenovirus 2	MAdV-2	mouse, rat	10
Parvoviridae	Parvovirus	Mouse parvovirus 1	MPV 1	mouse	100
		Mouse parvovirus 2	MPV 2	mouse	100
		Mouse parvovirus 3	MPV 3	mouse	10
		Mouse parvovirus 4	MPV 4	mouse	1000
		Mouse parvovirus 5	MPV 5	mouse	1000
		Minute virus of miceimmunosuppressive variant	MVMi	mouse	100
		Minute virus of mice prototype strain	MVMp	mouse	10
		Toolan’s H1 parvovirus	H-1 PV	rat	10
		Rat parvovirus	RPV	rat	1000
		Kilham rat virus	KRV	rat	10
		Rat minute virus	RMV	rat	10
Polyomaviridae	Polyomavirus	K-virus	K-virus	mouse, rat	1000
		Murine polyoma virus	MPyV	mouse	10
Muridae	Mus	*Mus musculus*			10
	Rattus	*Rattus rattus/norvegicus*			10

a determined in duplicates.

### Detection Limit (DL)

Ten-fold endpoint dilution series of plasmids containing the target virus sequence were prepared in the presence of 100 ng/µL murine DNA and analysed by rDVF. Despite the co-amplification and co-detection of murine gapdh and polA, DL ranged between 10 (for 14 species), 100 (for five species) and 1000 copies per PCR (for five species) (Table 1). The DL of the murine and rat DNA quality controls reached the level of 10 copies per PCR corresponding to about five cell equivalents.

### Specificity

rDVF was performed on 1×10^6^ copies of plasmid clones containing the respective target sequences diluted and stabilised in a background of 100 ng/µL murine or rat cellular carrier DNA or 20 ng/µL MS2RNA (Roche). Detection of all 27 targets (24 viruses and three controls) was highly specific (Table 2). MVMi and MVMp (prototype strain), *Rattus rattus* rhadinovirus (RratRHV) and *Rattus norvegicus* rhadinovirus (RnorRHV) as well as MPV2 and MPV4/MPV5 showed weak expected cross-reactivities due to high homology of the probe sequences with only two mismatches in their nucleotide sequence.

**Table 2:** Specificity of rDVF.

Family	PCR template	diluted in	Control-specific probes			Virus-specific probes																									
			Mus gapdh	Rat bg	PolA		RratRHV	RnorRHV	MmusRHV	RratCMV	MuHV-8	MuHV-2	MCMV	MuHV-4	ECTV	MAdV-1	MAdV-2	MPV1	MPV2	MPV3	MPV4	MPV5	MVMi	MVMp	H-1 PV	RPV	KRV	RMV	K-virus	MPyV	
Qualitycontrols	Mus gapdh	MS2RNA	**313** a	0	0		0	0	0	0	0	0	0	0	2	0	0	0	0	0	0	0	0	0	0	0	0	0	0	0	
	Rat bg	MS2RNA	0	**258**	0		0	0	0	0	0	0	0	0	0	0	0	0	0	0	0	0	0	0	0	0	0	0	0	0	
	PolA	MS2RNA	0	0	**217**		0	0	0	0	0	0	0	0	0	0	0	0	0	0	0	0	0	0	0	0	0	0	0	0	
Herpesviridae	RratRHV	murDNA^b^	425	0	150		**302**	94 c	0	0	0	0	0	0	0	0	0	0	0	0	0	0	0	0	0	0	0	0	0	1	
	RnorRHV	MS2RNA	0	0	2		0	**15**	0	0	0	0	0	0	0	0	0	0	0	0	0	0	0	0	0	0	0	0	0	0	
	MmusRHV	MS2RNA	0	0	0		0	0	**141**	0	0	0	0	0	0	0	0	0	0	0	0	0	0	0	0	0	0	0	0	0	
	RratCMV	murDNA	223	0	339		0	0	0	**598**	0	0	0	0	0	0	0	0	0	0	0	0	0	0	0	0	0	0	0	0	
	RCMV-E	ratDNA^d^	0	755	480		0	0	0	0	**1322**	0	0	0	0	0	0	0	0	0	0	0	0	0	0	0	0	0	0	0	
	RCMV-M	MS2RNA	0	0	0		0	0	0	0	0	**361**	0	0	0	0	0	0	0	0	0	0	0	0	0	0	0	0	0	0	
	MCMV	MS2RNA	0	0	0		0	0	0	0	0	0	**589**	0	0	0	0	0	0	0	0	0	0	0	0	0	0	0	0	0	
	MuHV-4	MS2RNA	0	0	0		0	0	0	0	0	0	0	**180**	0	0	0	0	0	0	0	0	0	0	0	0	0	0	0	1	
Poxviridae	ECTV	murDNA	270	0	177		0	0	0	0	0	0	0	0	**965**	0	0	0	0	0	0	0	0	0	0	0	0	0	0	0	
Adenoviridae	MAdV-1	murDNA	210	0	166		0	0	0	0	0	0	0	0	0	**766**	0	0	0	0	0	0	0	0	0	0	0	0	0	0	
	MAdV-2	murDNA	204	0	305		0	0	0	0	0	0	0	0	0	0	**102**	0	0	0	0	0	0	0	0	0	0	0	0	0	
Parvoviridae	MPV1	murDNA	216	0	154		0	0	0	0	0	0	0	0	0	0	0	**86**	0	0	0	0	0	0	0	0	0	0	0	0	
	MPV2a	murDNA	199	0	163		0	0	0	0	0	0	0	0	0	0	0	0	**508**	0	0	0	0	0	0	0	0	0	0	0	
	MPV3	murDNA	381	0	204		0	0	0	0	0	0	0	0	0	0	0	0	0	**233**	0	0	0	0	0	0	0	0	0	0	
	MPV4b	MS2RNA	0	0	0		0	0	0	0	0	0	0	0	0	0	0	0	504	0	**275**	0	0	0	0	0	0	0	0	0	
	MPV5a	MS2RNA	0	0	0		0	0	0	0	0	0	0	0	0	0	0	0	9	0	0	**640**	0	0	0	0	0	0	0	0	
	MVMi	murDNA	292	0	251		0	0	0	0	0	0	0	0	1	0	0	0	0	0	0	0	**727**	18	0	0	0	0	0	2	
	MVMp	MS2RNA	0	0	0		0	0	0	0	0	0	0	0	0	0	0	0	0	0	0	0	0	**293**	0	0	0	0	0	2	
	H-1 PV	murDNA	225	0	198		0	0	0	0	0	0	0	0	0	0	0	0	0	0	0	0	0	0	**94**	0	0	0	0	0	
	RPV	murDNA	206	0	167		0	0	0	0	0	0	0	0	0	0	0	0	0	0	0	0	0	0	0	**18**	0	0	0	0	
	KRV	murDNA	235	0	237		0	0	0	0	0	0	0	0	0	0	0	0	0	0	0	0	0	0	0	0	**159**	0	0	2	
	RMV	murDNA	213	0	142		0	0	0	0	0	0	0	0	0	0	0	0	0	0	0	0	0	0	0	0	0	**108**	0	0	
Polyomaviridae	K-virus	murDNA	401	0	363		0	0	0	0	0	0	0	0	0	0	0	0	0	0	0	0	0	0	0	0	0	0	**305**	0	
	MPyV	murDNA	347	0	321		0	0	0	0	0	0	0	0	0	0	0	0	0	0	0	0	0	0	0	0	0	0	0	**101**	

a specific MFI value.

b murine DNA.

c expected cross-reaction due to high sequence homology.

d rat DNA.

### Intra- and Interplate Reproducibility

Intra- and interplate variation of rDVF were analysed using two multi-target samples containing eight and nine DNA plasmids, respectively, in addition to murine and rat background DNA. Intraplate variation was calculated for these samples which were applied in duplicates to rDVF. The median CV of all probes within one plate was 10% (range 1–41%). Of the 100 expected positive reactions (two samples each in duplicates multiplied by 25 probes expected to be positive), only one reaction was negative resulting in a high reproducibility of 99% of positive signals despite the co-amplification of up to nine viruses and the cellular DNA.

Interplate variation was calculated analysing the same two multi-target samples on three plates in individual experiments at the same day. The median CV between the three plates was 21% (range 10–121%). Of the 150 expected positive reactions (two samples each on three plates multiplied by 25 probes), only one reaction was negative resulting in a reproducibility of 99.3% of positive signals.

### Direct Comparison of rDVF with Conventional PCR

Ten-fold dilution series of cell culture supernatants containing MVMi and MAdV-1, respectively, in unknown concentrations were used for a direct comparison of rDVF with conventional PCR used in the DKFZ routine health monitoring. While the sixth MVMi dilution step could be detected by the conventional PCR, rDVF was able to test positive the seventh dilution step indicating a 10-fold higher sensitivity of rDVF. rDVF was 1000-fold more sensitive for MAdV-1 (data not shown).

### Prevalence of DNA Viruses in 20 Mice and 16 Rats from Pet Shops

In a first feasibility study, rDVF was applied to mice and rats from pet shops which were suspected to show high levels of viral infections. DNA was extracted from homogenised organ tissues and ante-mortem materials that are used also in routine health monitoring. An animal was scored positive if at least one site (organ, ante-mortem sample) was positive for the respective virus. DNA quality controls were positive in 304 samples (96%), with the exception of 13 genital and pharyngeal swab samples.

**Figure 1:**
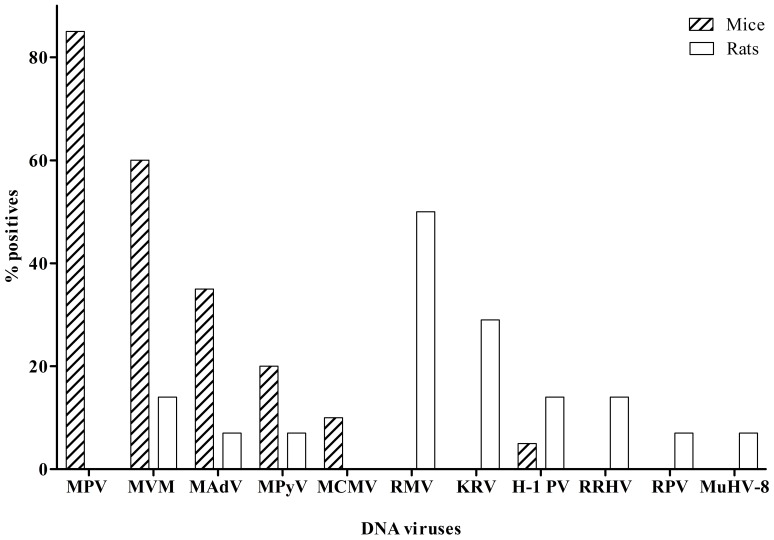
DNA viruses in pet shop rodents. Prevalence (y-axis) of DNA viruses (x-axis, categories) shown in mice (striped bars) and in rats (white bars). MPV1–5 is summarised as MPV, MVMi and MVMp as MVM and MAdV-1 and -2 as MAdV. RRHV summarises *Rattus rattus* rhadinovirus and *Rattus norvegicus* rhadinovirus.

Among the 20 mice, MPV1–5 was the most prevalent virus (85%), followed by MVMi/p (60%), MAdV-1/-2 (35%), MPyV (20%) and MCMV (10%) (Figure 1). Additionally, one unexpected hybridisation with the rat H-1 PV oligonucleotide probe was detected in one mouse. The presence of H-1 sequences in this sample was confirmed by sequencing of the PCR product. Moreover, multiple infections were observed in 89% of mice.

Most prevalent viruses in rats were RMV (50%), KRV (29%), H-1 PV and *Rattus rattus*/*Rattus norvegicus* rhadinovirus (RRHV) (each 14%), RPV, rat cytomegalovirus England strain (MuHV-8) and ECTV (each 7%) (Figure 1). Multiple infections were observed in 86% of rats. Furthermore, the detection of the mouse viruses MVM (detected in brain and thymus of two rat samples) and MPyV (detected in the brain sample of one rat) was confirmed via sequencing.

Parvoviruses like MPV, MVM, KRV and RMV were detected in liver, lung, kidney, spleen, intestinal tract including ileum and caecum, salivary gland, lymph nodes, trachea, but also in pharyngeal swabs and faeces. Once a mouse or rat was infected with the mentioned parvoviruses it was detected at least in two different organs. MAdV was mainly found in intestinal tract and mesenteric lymph nodes and MPyV in all analysed organs (Table S1).

### Comparison of rDVF and Charles River (CR) ELISA

Detection of viral infections in laboratory rodents is based mostly on indirect detection by serological analyses, e.g. ELISA. Although it is expected that indirect and direct detection techniques do not always lead to comparable results, rDVF was compared with commercial ELISA tests (Charles River). DNA extracted from different sites was analysed by rDVF, and sera from 20 mice and 14 rats from pet shops were analysed for antibodies by ELISA. The prevalence of 13 murine DNA viruses and 6 rat DNA virus infections detectable by both methods was examined. The CR ELISA was not able to specifically discriminate these 19 viruses, but grouped them in 11 groups. Consequently, the seven mouse parvoviruses MPV1–5 and MVMi/p were combined in MPV and MVM group, respectively, and the two mouse adenovirus species in the MAdV group resulting in a total of 11 rDVF DNA virus groups (Table 3).

**Table 3:** Detection of DNA virus groups in 20

Pet shop animals	DNA virus groups	rDVF:	+	−	−	+	Total	Kappa (CI.95)
		CR ELISA:	+	−	+	−		
Mice	MCMV		2	18	0	0	20	1 (0–1)
Mice	ECTV^a^		0	18	2	0	20	0 (0–1)
Mice	MAdV		6	9	4	1	20	0.5 (0.12–0.88)
Mice	MPV		15	3	0	2	20	0.69 (0.29–1)
Mice	MVM		7	7	1	5	20	0.42 (0.04–0.81)
Mice	K-virus		0	18	2	0	20	0 (0–1)
Mice	MPyV		2	15	1	2	20	0.48 (0–1)
Rats	ECTV^a^		0	13	0	1	14	0 (0–1)
Rats	MAdV		0	14	0	0	14	.^b^
Rats	H-1 PV		0	9	3	2	14	.^b^
Rats	RPV		1	5	8	0	14	0.08 (0–0.50)
Rats	KRV		4	10	0	0	14	1 (0–1)
Rats	RMV		7	2	5	0	14	0.29 (0–0.79)
**Mice and rats**	**Total**		**44**	**141**	**26**	**13**	**224**	**0.57 (0.45–0.70)**
Mice and rats	Poxviridae^c^		0	31	2	1	34	.^b^
Mice and rats	Adenoviridae^d^		6	23	4	1	34	0.61 (0.30–0.93)
Mice and rats	Parvoviridae^e^		34	36	17	9	96	0.46 (0.29–0.64)
Mice and rats	Polyomaviridae^f^		2	33	3	2	40	0.38 (0–0.89)

a CR ELISA is genus specific for orthopoxviruses but not ECTV species specific.

b this quantity cannot be calculated.

c ECTV.

d MAdV.

e MPV, MVM, H-1 PV, RPV, KRV and RMV.

f MPyV and K-virus.

Of the 224 possible reaction pairs (seven murine virus groups×20 mice and six rat virus groups×14 rats), 44 infections were concordantly positive (20%), 141 signals were concordantly negative (63%) and 39 discordant positive (17%) yielding in an overall kappa of 0.57 (CI.95 = 0.45–0.7) (Table 3). Of the 13 infections positive with rDVF only, eight were confirmed in a separated singleplex PCR followed by gel-electrophoresis and sequencing or by another serological test (multiplex serology, data not shown). Of the 26 infections positive by ELISA only, 11 were confirmed by additional serological tests (Immunofluorescence assay and/or multiplex serology, data not shown). The highest concordance was observed for MCMV, KRV and MPV with kappa values above 0.69 (Table 3).

In mice, MPV was the most prevalent virus (75% with CR ELISA and 85% with rDVF), followed by MVM (40% and 60%) and MAdV (50% and 35%). In rats RMV positivity (86% and 50%) was followed by KRV (29% and 29%).

## Discussion

Virus infections in laboratory rodents or contamination in biological materials including embryos, sera, monoclonal antibodies, cell lines, cell culture products, tissues and transplantable tumours might cause invalid and unreliable experimental results or lead to disease and even death in animals. Therefore, standardised programs are required assuring the quality of laboratory animals used in research [1]–[4]. These programs should include standardised monitoring of the animals themselves which is so far primarily done by serological assays (e.g. multiplexed fluorescence immune assay (MFIA), ELISA or immunofluorescence (IF)) but should also include standardised monitoring of biological materials which is usually done by molecular diagnostic techniques, occasionally also by time- and cost-intensive and animal-consuming M/RAP tests.

Serology is cheap and easy to perform, and only serum is needed to detect antibodies to all agents. However, serology does not detect exposure to a pathogen early in the cause of an infection and is inadequate for detecting exposure to agents in immunodeficient animals. Most importantly, positive results are not necessarily indicative of the continuing presence of the agent and do not enable definite conclusions to be made regarding the infectious potential of an animal.

Traditional molecular diagnostic techniques have the disadvantage of being time-intensive since they cover only a single type or a small group of rodent pathogens per reaction. By multiplexing, rDVF facilitates the simultaneous and specific detection of 24 DNA viruses and three DNA quality controls in one single PCR and one subsequent hybridisation reaction. No cross-reactivity was observed for any of the Luminex probes with unrelated amplimers. Besides specific probes, we also included universal parvovirus probes. Samples positive with the universal probes but negative with the parvovirus-specific probes could indicate the presence of unknown species for which no specific probe has been included. DL of rDVF ranged from 1 to 1000 copies per DNA virus. Investigation of the reproducibility revealed a high degree of robustness.

The 96-well format allows fast, simple and highly reproducible analyses of up to 500 samples in less than five days excluding DNA extraction. This offers the possibility to test biological materials fast and simple without the need of animals in contrast to the M/RAP test, but also offers the possibility to monitor the laboratory animals themselves and thus complementing or even replacing serological assays. If the detection of DNA viruses by rDVF is possible in ante-mortem material (faeces, swabs, urine), the number of sentinels could be reduced as well as the monitoring in individually ventilated cages (IVC) could be improved.

The detectability of DNA viruses in organic materials by rDVF was validated with animals bought in pet shops, having a higher infection rate than laboratory mice and rats. Since PCR methods for the detection of DNA viruses are not primarily a routine part of health monitoring program at the DKFZ, comparison of rDVF was done with the CR ELISA tests detecting antibodies in sera against viral antigens. Concordant results could be achieved in 83% of all 224 possible infections. Of the 39 discordant results, 67% were observed within the parvovirus family. The presence of a so far unknown related species could induce cross-reactions with either the integrated parvovirus probes or the integrated parvovirus antigens and hence result in the discrepant identification of parvovirus species. Confirmatory, sequencing of 6 rDVF parvovirus infections where the expected pattern was not observed (i.e. reactivity with universal plus specific parvovirus probes) but cross-reactivity with RMV or MPV3 only revealed a so far unknown parvovirus species (unpublished observation). It remains to be seen, whether this species may be relevant for laboratory animals as well. In contrast to rDVF, the antigen for ectromelia included in CR ELISA detects most likely antibodies to all orthopoxviruses. Consequently, the two discrepant ectromelia infections in mice may be infections by other poxviruses, e.g. cowpox [36]. Moreover, discrepancies observed between both methods, e.g. for MAdV, might be explained by the fact that naturally occurring variants or related virus species are better detectable by serological assays. In addition, acute infections can only be identified by molecular test whereas past infections are detectable by serological tests. Furthermore, viruses can be restricted to certain organs. If these organs are not included as sample material, rDVF may lead to false-negative results.

Parvoviruses have been found in organs containing rapidly dividing cells [1], [37], [38]. In consistence, parvoviruses were detected mostly in kidney, spleen, mesenterial lymph nodes, lung, liver and intestinal tract by rDVF (Table S1). As described in the literature, MPyV was found in lymph nodes [39], MCMV in spleen [40], MAdV in lungs and intestine [41] by rDVF. The analysis of ante-mortem samples (e.g. faeces and genital, dermal or nose swabs) is discussed to be sufficient for the detection of the majority of DNA viruses included in rDVF. Our data show that the application of ante-mortem material in rDVF seems to be possible, however, with a slightly reduced sensitivity compared to organ material. However, this is based on small numbers of ante-mortem material and has to be confirmed in larger studies.

Unpublished data comparing rDVF with M/RAP test revealed an excellent negative predictive value of rDVF, while the positive predictive value could not be assessed due to the low prevalence of contamination. Consequently, rDVF may not only be used for detecting DNA viruses in animals but also in embryonic stem cells, tumour cells and feeder cells. However, in 2 (7%) cases DNA sequences typical for RMV were discovered in murine embryonic stem cell lines by rDVF only. These infections were not detected by the initial MAP test. Sequencing of the amplimers confirmed the presence of RMV including three mismatches between forward primer and probe. Therefore, our data showed that murine material may be contaminated by rat viruses or unknown mouse viruses. Moreover, the data confirm that so far unknown viruses may exist not only in pet shop animals but also in laboratory settings. In addition, newly identified virus species should be implemented in routine screening.

rDVF will become an integral part of laboratory animal quality assurance program of the DKFZ, supplementing or replacing traditional serology, bacteriology, virology and pathology techniques as it is time- and cost-efficient, and would reduce the number of animals needed. Furthermore, acute infections and infections leading to insufficient antibody production (e.g. in immunocompromised animals, variation in sensitivity to infection between strains) may be missed by serology and better detected by PCR. To complete the relevant pathogen panel as suggested by the FELASA, we are currently developing two additional high-throughput assays detecting RNA viruses and bacteria in laboratory rodents. In conclusion, rDVF appears to be a powerful tool in the assurance of laboratory animal quality and may simplify and improve routine health monitoring.

## Supporting Information

Table S1 Detected DNA viruses in different organs of pet shop animals.
(DOC)
