# Supplementary material for: A Bead-Based Multiplex Assay for the Detection of DNA Viruses Infecting Laboratory Rodents
Source: PLoS One. 2014 May 16;9(5):e97525. doi: 10.1371/journal.pone.0097525 (PMC4023972; doi:10.1371/journal.pone.0097525)
Supplement: Table S1 — Detected DNA viruses in different organs of pet shop animals. (DOC) [file pone.0097525.s001.doc]

**Supporting Information**

**Table S1.** Detected DNA viruses in different organs of pet shop animals.

^a^ includes caecum, ileum and stomach

^b^ not tested

^c^ no pathogen detected
